# Factors associated with emergency room readmission after elective surgery for ovarian carcinoma

**DOI:** 10.1186/s12905-023-02579-7

**Published:** 2023-09-04

**Authors:** Rosa A. Salcedo-Hernandez, Salim Barquet-Muñoz, David Isla-Ortiz, Florencia Lucero-Serrano, Leonardo S. Lino-Silva, David Cantú de León, Lucely Cetina-Perez

**Affiliations:** 1 Programa de Maestría y Doctorado en Ciencias Médicas, Odontológicas y de la Salud. UNAM, Mexico City, Mexico; 2 Departamento de Ginecología, Instituto Nacional de Cancerología, Mexico City, Mexico; 3 Departamento de Patología, Instituto Nacional de Cancerología, Mexico City, Mexico; 4 Dirección de Investigación, Instituto Nacional de Cancerología, Mexico City, Mexico; 5 Subdirección de Investigación Clínica, Instituto Nacional de Cancerología, Mexico City, Mexico; 6 Clinical Research, Instituto Nacional de Cancerología, San Fernando 22, 14050 ZP Mexico City, Mexico; 7 Division of Research, Instituto Nacional de Cancerología, San Fernando Avenue 22, 14050 ZP Mexico City, Mexico

**Keywords:** Ovarian carcinoma, Surgery, Emergency room, Complications

## Abstract

**Background:**

Hospital readmission is a quality metric of hospital care and has been studied in ovarian carcinoma, but its evaluation has several limitations. Also, emergency room (ER) readmission is considered an adverse effect because it represents patient costs. Therefore, our objective was to determine the rate of ER readmission, its causes, and associated factors.

**Methods:**

A retrospective study of 592 patients with ovarian carcinoma who underwent upfront surgery, neoadjuvant therapy, or surgery for recurrent disease. An analysis of factors associated with ER readmission, hospital readmission, and surgical complications was performed, including multivariate analysis to assess for case-mix factors.

**Results:**

Of 592 patients, the median age was 51 years, and the predominant type of treatment was the neoadjuvant approach (52.9%); 46% underwent upfront surgeries and six surgeries for recurrence. The ratio to ER readmission was 11.8% (70 patients), of whom 12 patients were admitted more than once. The factors associated with ER readmission were prolonged surgery, intraoperative bleeding, extended hospital stay, the time of the day when the surgery was performed, and post-surgical complications. The hospital readmissions were 4.2%, and the overall morbidity was 17.6%. In the multivariate analysis, the only factor associated with ER readmission was the presence of surgical complications (OR = 39.01). The factors independently associated with hospital readmission were the entrance to the intensive care unit (OR = 1.37), the presence of surgical complications (OR = 2.85), and ER readmission (OR = 1.45).

**Conclusion:**

ER readmission is an adverse event representing the presence of symptoms/complications in patients. Evaluating the ER readmission independently of the readmission to the hospital is critical because it will allow modifying medical care behaviors to prevent patients from unnecessarily returning to the hospital after a hospital discharge to manage preventable medical problems.

**Trial registration:**

researchregistry7882.

## Highlights

Emergency room readmission is an adverse effect of ovarian carcinoma and represents health system costs.

Readmission was associated with prolonged surgery, intraoperative bleeding, extended hospital stays, and surgeries performed in the afternoon.

Emergency room readmission is strongly associated with hospital readmission.

Rosa A. Salcedo-Hernandez, David Cantú-de León and Lucely Cetina-Perez have contributed equally to the present study.

## Background

Ovarian carcinoma is considered a public health problem; according to Globocan 2020, it is ranked eighth in incidence and mortality in women [1]. Treatment for ovarian carcinoma is multidisciplinary, and the surgery is integral to the treatment, either from the beginning or during the disease course [2]. International organizations have developed indicators to measure the quality of surgeries, including the systematic recording of complications as an outcome indicator [3].

Hospital readmission has been considered a quality metric of hospital care. It has been a focus of interest for a long time, and in the United States, a program to reduce readmissions has been created and applied to several diseases [4, 5]. This indicator has been proposed to represent the quality of care for postoperative patients with ovarian carcinoma; however, it possesses disadvantages: (a) does not consider the type of population to which it is being applied (patients with advanced disease, fragile, etc.), (b) increase in readmissions can be due to the extension of surgery (in particular, during primary surgery) and, (c) the use of neoadjuvant chemotherapy can be inadvertently favored as the primary treatment in advanced stages to avoid the possible complications of extensive surgery and possible hospital readmissions [6, 7].

Emergency room (ER) readmission (does not mean hospital readmission because many patients attend ER services for several reasons: doubts about their care, mild pain, etc., which does not necessarily imply that the patient is hospitalized) is considered an adverse effect of medical care because it represents a return to the hospital due to the presence of symptoms or complications. This ER readmission means costs for patients regarding time, trips to/from the hospital, loss of work opportunities, and out-of-pocket expenses; it also is a burden for primary caregivers. In addition, it also represents an expense for the health system [8].

A 12% rate of ER readmission after surgery for gynecological cancer has been reported [8, 9]. The factors associated with hospital readmission – which could also be associated with ER readmission after discharge – are poor communication with the patient, insufficient follow-up after discharge, and lack of coordination between the different levels of hospital care [8]. However, there is no evidence in the literature regarding the associated factors and causes of ER readmission for postoperative patients with ovarian carcinoma; although factors associated with hospital readmission have been described, the factors for ER readmission could be different because hospitalization is not required for their resolution.

This study aimed to determine the ER readmission rate, analyzing the consequent rate of hospital readmission, its causes, and associated factors in patients with ovarian carcinoma.

## Methods

This is a retrospective study in which 592 patients with ovarian carcinoma were treated in our institution with either elective upfront surgery, neoadjuvant chemotherapy, or surgery for recurrence from 2011 to 2017. The patients were at least 18 years old and treated for ovarian carcinoma (any histological subtype). We collected demographic data and the following variables: clinical stage, type of treatment (upfront surgery, neoadjuvant approach, or surgery for recurrence), surgical variables (duration of surgery, bleeding, need for transfusion, stay in intensive care, and complexity of surgery) that influence the presence of postsurgical complications. In addition, the proportion of hospital readmissions, ER readmissions, surgical reoperations, and surgical complications were calculated. Postsurgical complications were categorized according to the Clavien–Dindo classification [10]. Defining whether surgical complications are intraoperative or postoperative is important because each has different implications. Intraoperative complications are essential to define since they relate to the surgery’s and surgical technique’s complexity. It is necessary to identify and repair them immediately since these can be so serious that they directly threaten the patient’s life or determine the risk of future postoperative complications.

In many places, postoperative complications are quality indicators that reflect a late recovery, prolong the hospital stay, are risk factors for the patient to return to seek medical attention at an emergency department, or warrant hospital readmission.ER readmission was defined as the admission of the patient to the ER within 30 days after discharge for ovarian carcinoma surgery, according to previous publications [8]. Hospital readmission was defined as the patient’s hospitalization within 30 days after discharge [4]. Planned readmissions (administration of chemotherapy, port catheter placement, etc.) were not considered. Mortality at 30 days was defined as any death that occurred during the 30 days after elective surgery for ovarian carcinoma [11].

Grading the complexity of the surgery was established according to a previously published score as follows [12]: 0–3 = low, 4–7 = intermediate, and 8 + = high. Cytoreduction was considered either (a) suboptimal (residual disease > 1 cm) or (b) optimal (no macroscopically visible residual tumor) based on the last published recommendations [13].

This study was approved by the ethics and research committee of our institution (registration number (018/031/GII) (CEI/1,282,718)).

By the journal’s guidelines, we will provide our data for the reproducibility of this study in other centers upon reasonable request.

### Statistical analysis

The variables were analyzed based on the data distribution, determined with the Kolmogorov–Smirnov test. First, the proportions of surgical complications, hospital readmissions, and ER readmissions were calculated, and the causes of these outcomes were assessed. Subsequently, an analysis of the factors associated with ER readmission, hospital readmission, and presence of complications was performed, stratified by type of surgery using the chi-square test for nominal variables and the Mann-Whitney’s U test for nonparametric data.

Logistic regression was used to determine the factors independently associated with ER readmission, hospital readmission, and surgical complications. SPSS 24.0 was used for the statistical analysis.

## Results

### Population

Of 592 patients, the median age was 51 years, with an interquartile range (IQR) of 44–59 years. 20.2% (120 patients) were illiterate, and 47% (279 patients) had primary education. As seen in Table 1, the median age varied depending on the type of surgery, and there were differences in length of surgery and intraoperative bleeding, with the latter being higher among patients who underwent primary surgery.

**Table 1:** Demographic and surgical variables according to the type of surgery

Variable	Upfront surgery (n = 273)	Neoadjuvant therapy + surgery (n = 313)	Recurrence ( n = 6)	*p* ^**^
Age — Median (IQR)	49 (43–56)	52 (45–60)	63 (57–68)	< 0.001
Time to surgery*(IQR)	34 (24–45)	35 (24–47)	26 (20–30)	0.114
Length of surgery (min)— median (IQR)	200 (120–265)	155 (105–235)	225 (80–370)	< 0.001
Blood loss (mL) — Median(IQR)	300 (100–600)	200 (100–350)	175 (100–250)	< 0.001
Hospital stay (days) — Median (IQR)	2 (1–3)	2 (1–3)	3 (2–5)	0.386

IQR = interquartile range, min = minutes

* Time elapsed between treatment decision and surgery in days

** Mann-Whytney U test

The most predominant type of treatment was the neoadjuvant approach (52.9%, 313 patients); 46% (276 patients) underwent upfront surgeries and six surgeries for recurrence. Half of these procedures were performed during the morning shift (08:00 to 14:00). Table 2 shows the clinical stage of the disease at the time of diagnosis and the factors concerning surgical complexity, with primary surgeries involving greater complexity (34.1% were of intermediate complexity) and resulting in a higher proportion of intraoperative complications (11.7%).

**Table 2:** Surgical variables of 586 patients with ovarian carcinoma*

Variable	Upfront surgery n (%)	Neoadjuvant therapy + surgery n (%)	p**
Stage Early Advanced	111 (40.7) 162 (59.3)	4 (1.3) 309 (98.7)	< 0.001
Cytorreduction Complete/optimal Suboptimal	222 (81.3) 51 (18.7)	248 (79.2) 65 (20.8)	0.527
Participation in clinical trial No Yes	251 (91.9) 22 (8.1)	276 (88.2) 37 (11.8)	0.131
Intensive Care Unit admission No Yes	261 (95.6) 12 (4.4)	298 (95.2) 15 (4.8)	0.819
Intraoperative complications No Yes	241 (88.3) 32 (11.7)	297 (94.9) 16 (5.1)	0.011
Surgical complexity Low Intermediate High	178 (65.2) 93 (34.1) 2 (0.7)	261 (83.4) 52 (16.6) 0	< 0.001
30-day readmission No Yes	261 (95.6) 12 (4.4)	298 (95.8) 13 (4.2)	0.867
Emergency room readmission No Yes	240 (87.9) 33 (12.1)	276 (88.2) 37 (11.8)	0.663
Surgical reintervention No Yes	265 (97.1) 8 (2.9)	299 (95.8) 13 (4.2)	920
Death ay 30 days No Yes	270 (98.9) 3 (1.1)	310 (99.4) 2 (0.6)	0.550
Surgical complications No Yes	223 (81.7) 50 (18.3)	260 (83.1) 53 (16.9)	0.661

*The 6 surgeries during recurrence were excluded due to the small number of procedures and that their comparison would not be valid

**Chi square, in cases where there were events less than 5, Fisher’s exact was used

### ER readmission and causes

The proportion of patients readmitted to the ER, regardless of the type of surgery, after their hospital discharge and before their first postoperative follow-up consultation was 11.8% (70 patients), of whom 17.1% (12 patients) were admitted more than once. Of patients who returned to ER (70 patients), 70% did so during the first two weeks after hospital discharge. The leading causes of ER readmission were abdominal/wound pain in 23 (32.8%) cases, wound dehiscence, seroma, or infection in 13 (18.5%) cases, gastrointestinal disturbances in 11 (15.7%) cases, drug-related issues in 5 (7.1%) cases, drains and probes dysfunction in 6 (8.5%) cases, and other causes in 12 patients (17.1%).

The patient’s treatment in the ER was medication adjustment or additional medical management (analgesic or antibiotic), instruction for wound management, treatment of alarm symptoms, and drainage care (Table 3).

**Table 3:** Factors associated with readmission to the emergency department of 592 patients postoperatively for ovarian carcinoma

	Emergency room readmission		
	No	Yes	p^*^
Age — Median (IQR)	51 (44–59)	51 (43–57)	0.476
Time to surgery^+^(— Median(IQR)	34 (24–46)	33 (23–45)	0.339
Length of surgery (min)— Median (IQR)	170 (108–245)	210 (150–270)	0.006
Blood loss (mL) — Median (IQR)	200 (100–400)	350 (200–600)	0.001
Hospital stay (days) — Median (IQR)	2 (1–3)	2 (2–4)	0.003
Stage (n, %) Early Advanced	104 (19.9) 418 (80.1)	11 (15.7) 59 (84.3)	0.403
Cytorreduction (n,%) Complete/optimal Suboptimal	422 (80.8) 100 (19.2)	54 (77.1) 16 (22.9)	0.464
Time of the surgery (n, %) Morning Afternoon	306 (58.6) 216 (41.4)	31 (44.3) 39 (55.7)	0.023
Participation in clinical trial (n, %) No Yes	469 (89.8) 53 (10.2)	64 (91.4) 6 (8.6)	0.678
Intensive care unit admission (n, %) No Yes	495 (94.8) 27 (5.2)	68 (97.1) 2 (2.9)	0.399
Intraoperative complications (n, %) No Yes	478 (91.6) 44 (8.4)	66 (94.3) 4 (5.7)	0.639
Surgical complexity (n, %) Low Intermediate High	391 (74.9) 129 (24.7) 2 (0.4)	52 (74.3) 18 (25.7) 0	0.910
Type of treatment (n, %) Surgery Neoadjuvant therapy + surgery Surgery for recurrence	240 (46) 276 (52.9) 6 (1.1)	33 (47.1) 37 (52.9) 0	0.663
Surgical complications (n, %) No Yes	473 (90.6) 49 (9.4)	15 (21.4) 55 (78.6)	< 0.001

IQR = interquartile range, min = minutes

*Mann-Whitney U, Chi-square or Fisher’s exact according to the number of events

+ Time elapsed between treatment decision and surgery

### Hospital readmission

The proportion of hospital readmissions was 4.2% (25 patients). The leading causes of hospital readmission were infection of the surgical wound (9 patients), gastrointestinal disorders (9 patients), evisceration (3 patients), and other complications in four patients. Table 4 shows the factors associated with hospital readmission.

**Table 4:** Factors associated with hospital readmission

	No-readmission	readmission	p^*^
Age — Median (IQR)	51 (44–59)	53 (45–56)	0.997
Time to surgery^**+**^**(**Median (IQR)	35 (24–46)	25 (21–36)	0.023
Length of surgery (min)— Median (IQR)	170 (110–245)	215 (160–300)	0.038
Blood loss (mL) — Median (IQR)	200 (100–450)	300 (200–600)	0.067
Hospital stay (days) — Median (IQR)	2 (1–3)	3 (1–6)	0.026
Time to first visit— Median(IQR)	9 (7–11)	9 (7–11)	0.706
Stage (n, %) Early Advanced	110 (19.5) 455 (80.5)	5 (20) 20 (80)	0.948
Cytorreduction (n,%) Complete/optimal Suboptimal	456 (80.7) 109 (19.3)	19 (76) 6 (24)	0.560
Type of treatment (n, %) Surgery Neoadjuvant therapy + surgery Surgery for recurrence	261 (46.2) 298 (52.7) 6 (1.1)	12 (48) 13 (52) 0	0.867
Time of the surgery (n, %) Morning Afternoon	321 (56.8) 244 (43.2)	14 (56) 11 (44)	0.936
Participation in clinical trial (n, %) No Yes	511 (90.4) 54 (9.6)	20 (80) 5 (20)	0.089
Intensive care unit admission (n, %) No Yes	541 (95.8) 24 (4.2)	20 (80) 5 (20)	< 0.001
Intraoperative complications (n, %) No Yes	519 (91.9) 46 (8.1)	23 (92) 2 (8)	0.980
Surgical complexity (n, %) Low Intermediate High	422 (74.7) 141 (25) 2 (0.4)	19 (76) 6 (24)	0.950
Emergency room admission (n, %) No Yes	512 (90.6) 53 (9.4)	8 (32) 17 (68)	< 0.001
Surgical reoperation (n, %) No Yes	555 (98.2) 10 (1.8)	14 (56) 11 (44)	< 0.001
Surgical complications (n, %) No Yes	486 (86) 79 (14)	0 25 (100)	< 0.001

IQR = interquartile range, min = minutes

*Mann-Whitney U, Chi-square or Fisher’s exact according to the number of events

+Time elapsed between treatment decision and surgery

### Mortality

The morbidity rate for the 592 postoperative patients was 17.6% (104 patients), with 37.4% (39 patients) having grade III and IV morbidity. The main grade III and IV complications were intestinal leakage/occlusion in 11 (27.7%) patients, evisceration, dehiscence, and infection of the wound in 10 (25%) cases, bleeding/shock in 6 (16.6%) cases, abdominal/pelvic abscesses in 4 (11.3%), and other causes in 8 (19.4%). In addition, the rate of surgical reoperation at 30 days was 3.5% (21 patients).

### Analysis of factors associated with readmission

The factors associated with surgical complications were duration of surgery, intraoperative bleeding, and hospital stay (Table 5). In the multivariate analysis, the only factor independently associated with ER readmission was the presence of surgical complications (OR = 39.01, CI = 20.05–76.24). The variables independently associated with hospital readmission were ICU admission ICU (OR = 1.37, CI = 1.15–2.56), the presence of surgical complications (OR = 2.85, CI = 1.59–4.9), and ER readmission (OR = 1.45, CI = 1.11–1.80).

**Table 5:** Factors associated with the presence of surgical complications

	No	Yes	P^*^
Age — Median (IQR)	51 (44–58)	53 (43–59)	0.764
Time to surgery^+^ (Median (IQR)	35 (25–46)	32 (21–45)	0.145
Length of surgery (min)— Median (IQR)	170 (105–240)	215 (150–283)	< 0.001
Blood loss (mL) — Median (IQR)	200 (100–400)	325 (200–600)	< 0.001
Hospital stay (days) — Median (IQR)	2 (1–3)	2 (2–5)	< 0.001
Time to first appointment— Median (IQR)	9 (7–11)	9 (7–10)	0.744
Stage (n, %) Early Advanced	97 (19.9) 391 (80.1)	18 (17.3) 86 (82.7)	0.548
Cytoreduction (n, %) Complete/optimal Suboptimal	397 (81.4) 91 (18.6)	79 (76) 25 (24)	0.208
Time of the surgery (n, %) Morning Afternoon	285 (58.4) 203 (41.6)	52 (50) 52 (50)	0.116
Participation in clinical trial (n, %) No Yes	441 (90.4) 47 (9.6)	92 (88.5) 12 (11.5)	0.555
Intensive care unit admission (n, %) No Yes	466 (95.5) 22 (4.5)	97 (93.3) 7 (6.7)	0.340
Intraoperative complications (n, %) No Yes	451 (92.4) 37 (7.6)	93 (89.4) 11 (10.6)	0.310
Surgical complexity (n, %) Low Intermediate High	372 (76.2) 114 (23.4) 2 (0.4)	71 (68.3) 33 (31.7) 0	0.168
Type of treatment (n, %) Surgery Neoadjuvant therapy + surgery Surgery for recurrence	223 (45.7) 260 (53.3) 5 (1)	50 (48.1) 53 (51) 1 (0.9)	0.907

IQR = interquartile range, min = minutes

+ Time elapsed between treatment decision and surgery in days

*Mann-Whitney U, Chi-square or Fisher’s exact according to the number of events

## Discussion

The objective of this study was to measure ER readmission and hospital readmission after surgery for ovarian carcinoma and its causes or associated factors. Among 592 patients, 11.8% were readmitted to the ER, and the most common reasons were pain, wound complications, and gastrointestinal alterations. In addition, we found that the factors associated with ER readmission were more prolonged duration of surgery, more significant intraoperative bleeding, surgery during the afternoon shift, and postoperative complications.

Surgery for ovarian cancer is a fundamental part of the primary treatment of this neoplasm, which has evolved, increasing radicality (upper abdomen approach) to leave no residual disease (it is one of the independent factors of survival); Due to the above, efforts have been made to establish guidelines or consensus on the perioperative management of these patients to reduce morbidity or mortality and allow systemic treatment not to be delayed [14]. The importance of surgery remains even after the introduction in recent years of new therapeutic options and personalized treatment, such as PARP inhibitors, which have shown a benefit in the survival of patients with ovarian cancer when used in the primary stage, mainly in those that responded to systemic treatment with platinum and cytoreductive surgery could be performed [15].

Due to this importance, It is necessary to identify and avoid outcomes such as the recurrence of symptoms or occurrence of complications that decrease quality of life and increase costs, which can have a great impact on patients with frailty (a clinical syndrome that increases the vulnerability of the patient) which is common to find in patients with ovarian cancer, which not only increases the probability of complications but also of death [16, 17]. Various outcomes have been widely described, including the percentages of complete cytoreduction, complications, hospital readmissions, initiation of chemotherapy after surgery, disease-free period, and overall survival [13, 18].

ER readmission has been reported as an adverse event of medical care and an outcome that does not represent hospital readmission but does represent the presence of symptoms, probable complications, and expenses for both the patient and the health system. In our study, 11.8% of patients were readmitted to ER, and 7.1% were admitted on more than one occasion, which could indicate that medical issues were not fully resolved before discharge or after the first ER visit, requiring a return for further care. Our readmission rate is similar to that reported in the literature (12%) after surgery for gynecological cancer [8, 9, 17]. However, our data were obtained from medical records; therefore, it is unknown if the patients went to the emergency room at another hospital or medical service, and the rate of ER readmission could be underestimated.

Our study’s leading causes of ER readmission were abdominal/wound pain, wound complications, and gastrointestinal alterations. Compared with the leading causes of hospital readmission, those for ER readmission were different, could be managed on an outpatient basis, and did not require hospitalization. Most patients who sought care for abdominal pain only required analgesic adjustment, and 7.1% sought care for doubts or adjustments related to medications, suggesting that these readmissions could have been avoided. These findings reinforce that the transition from hospital to home is a vulnerable period for patients; poor communication with patients and their primary caregivers and insufficient follow-up could result in the deterioration of the patient’s health status or in need to return to the hospital to resolve symptoms and doubts, adjust medications, or be readmitted to the hospital [19].

Factors associated with ER readmission were more prolonged surgery, more significant intraoperative bleeding, the time of day when the surgery was performed (more frequent surgeries during the afternoon), postsurgical complications, and hospital stay. All these factors have already been associated with a decrease in the possibility of cytoreduction [20] and an increase in difficulties in the weeks after surgery and, therefore, the search for medical care, resulting in either hospital readmission [21] or ER admission. However, the multivariate analysis results indicated that only complications were associated with ER readmission, which is expected because the presence of signs or symptoms will cause patients to seek medical attention. In addition, as mentioned above, not all patients required hospitalization and were managed on an outpatient basis. However, the study’s retrospective nature, with possible selection bias, may have affected our results and not accounted for already known associated factors; therefore, a prospective analysis would help identify risk factors and establish several prevention or management strategies.

A total of 70% of the patients who sought care at ER did so during the first two weeks after hospital discharge, indicating that this is a vulnerable period for patients when the presence of unresolved medical problems or worsening and/or appearance of symptoms make it necessary to seek medical attention. In addition, as mentioned above, it is the period when the continuity of treatment is the responsibility of the patient and the primary caregiver. If there is an adequate transition or follow-up, there may be improvements [16]. Harel Z et al. reported that the median time to ER readmission was 11 days (IQR 5–19 days). Although the population in this study was not oncological, the results indicate the vulnerability of patients after hospital discharge [22]. Wang et al. reported that 33% of patients sought care at ER after the first week after discharge; extended hospital stay was among the associated factors, a factor that was also reported in our population [8].

The rate of hospital readmissions in our series was 4.2%, indicating that our unplanned readmissions were low and that the patient’s symptoms and medical problems were managed on an outpatient basis. This percentage is within the range reported in the literature (2.5–19.3%)^6^. Although there are contradictory opinions about whether this outcome should be used as an indicator of quality, [4, 5, 23] this outcome should be monitored to avoid an increase in readmissions. The leading causes of hospital readmission were surgical site pain, wound infection, and grade III and IV gastrointestinal alterations, findings that are consistent with the literature [4] and that indicate the need for greater intrahospital and post-discharge wound care. The independent factors associated with hospital readmission were admission to the UCI, ER readmission, and surgical reintervention, which suggest more complex procedures that required closer patient care and complications that required another surgery. Although our readmission rate is low concerning that for other series, the retrospective nature of the study and the selection of patients (younger and low and intermediate complexity surgeries) could explain this low proportion of readmissions [21]. A prospective cohort could provide more robust data to determine points of improvement in care.

The overall morbidity in our patients was 17.6% (104 patients); of these, 37.4% (39 patients) had grade III and IV morbidity. Thus, there was a low proportion of patients with severe morbidity, which is explained in our study by the type of patients, the complexity of the procedures that were performed, and the elective nature of the procedures. The morbidity in our research is like that reported in the literature for patients younger than 50 years [24]. The morbidity-related factors were longer hospital stays, postoperative bleeding, and longer surgery duration, representing greater surgical complexity. Aletti et al. [12] reported that greater surgical complexity and poor nutritional and functional status are associated with morbidity within the first 30 days after surgery, consistent with our results.

Many readmissions to the emergency department can be preventable, and better communication with patients and primary caregivers at the time of discharge could prevent ER readmission because, most of the time, the care provided was only medication adjustments or the provision of instructions for wound care and drainage management. With all the information generated in our study and with what has been published by other authors [19], improving the transition from in-hospital to out-of-hospital care, either with interventions provided by a medical team and/or through training provided to patients and primary caregivers, would reduce, or could avoid ER readmissions.

The main limitation of our study is its retrospective nature, with the consequent selection bias. In addition, the data were obtained from electronic files, which, although reliable, may lack precision in some categories and lack information in others, such as if the patients went to another health institution or private doctor in search of medical attention. These factors would lead to an underestimation of our results.

The strength of our study is that we focused on an adverse event that is not frequently reported (ER readmission). Although this outcome does not represent hospital readmission, it should be a focus of attention due to complications and frailty in patients and the associated costs for patients and the health service.

## Conclusions

ER, readmission is an adverse event representing the presence of symptoms/complications in patients. The proportion of patients readmitted to the ER was 11.8%, and the leading cause was wound complications. The ratio of hospital readmissions was 4.2%. The only factor independently associated with ER readmission was the presence of surgical complications. Factors associated with hospital readmission were ICU admission, surgical complications, and ER readmission. ER evaluation independently of hospital readmission is essential because it will allow for modified conduct of medical care to prevent patients from returning to the hospital to seek care.

## Data Availability

This was a retrospective study, and after Institutional Review Board (IRB) approval, the data were obtained from the clinical files of patients with ovarian carcinoma. The data analyzed in this study are available directly to the authors and for a good purpose. Any request can be sent to Rosa Salcedo-Hernández ( rosasalher@gmail.com).

## Declarations

This study was conducted in accordance to the Declaration of Helsinki (2013), the Good Clinical Practice, and the General Health Law in Research Matters of México.

Because all the information was obtained from the clinical charts, an specific informed consent for this study was waived by Comité de Etica en Investigación, which is the intitutional ethics committee.
